# Untargeted metabolomic analysis of date seed oil (*Phoenix dactylifera* L.) using UHPLC-ESI-QTOF-MS - evaluation of the geographical origin effect

**DOI:** 10.1016/j.fochx.2025.103162

**Published:** 2025-10-14

**Authors:** Said El Harkaoui, Katharina N'Diaye, Zoubida Charrouf, Sascha Rohn, Stephan Drusch, Bertrand Matthäus

**Affiliations:** a Max Rubner-Institut, Federal Research Institute for Nutrition and Food, Department for Safety and Quality of Cereals, Schützenberg 12, 32756 Detmold, Germany; b Department of Food Chemistry and Analysis, Institute of Food Technology and Food Chemistry, Technische Universität Berlin, Berlin, Germany; c Department of Food Technology and Food Material Science, Institute of Food Technology and Food Chemistry, Technische Universität Berlin, Berlin, Germany; d Department of Chemistry, Faculty of Sciences, Mohammed V University in Rabat, Morocco

**Keywords:** *Phoenix dactylifera*, date seed oil, metabolomic, geographical origin, authenticity, multivariate analysis, UHPLC-ESI-QTOF-MS

## Abstract

The chemical composition of edible oils is influenced by geographical origin, making traceability essential for ensuring authenticity and quality, particularly for unconventional (edible) oils like date seed oil (DSO). For economic, food safety, and research purposes, this study aimed to characterize the metabolomic profile of DSO and assess the impact of Moroccan origin to identify key chemical markers linked to regional differences. An untargeted metabolomic approach using UHPLC-QTOF-MS was applied to DSO samples from three Moroccan palm groves: *Allougoum*, *Alnif*, and *Errachidia*. PCA revealed modest clustering for *Allougoum* samples, while OPLS-DA enabled selection of 50 features contributing to differentiation. Among these, 25 metabolites were tentatively identified as geographical markers with three, n-(3-oxohexanoyl) homoserine lactone, indole-3-carboxaldehyde, and vanillin, confirmed using authentic standards. Hydroxy fatty acids were the most represented class, with four compounds tentatively annotated. This study provides the first comprehensive metabolomic profile of DSO and highlights potential markers for origin differentiation.

## Highlights

• Metabolomic profile of date seed oil (DSO) varies by region.
• Chemometrics showed differences and highlighted key features forming clusters.
• 25 features could be annotated with hydroxy fatty acids as dominant chemical class.
• Findings support region-specific labels for DSO.

## Graphical abstract

**Figure:**
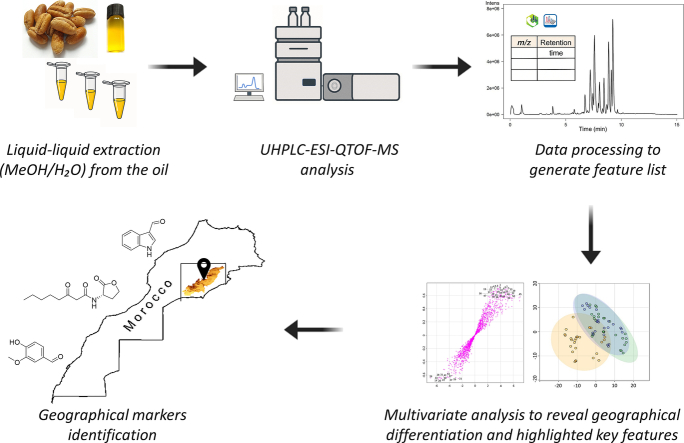

## 1 Introduction

The date palm (*Phoenix dactylifera* L.) is a crucial crop in arid and semi-arid regions, especially in North Africa and the Middle East, where its fruits serve as a major economic and nutritional resource. In Morocco, it is the most important arboriculturally in arid areas, playing a vital role in both traditional and commercial agriculture (Bouhlali, Alem, et al., 2017). While date fruits are commonly processed into products such as syrup, jam, and vinegar, their seeds, so far regarded as waste, are increasingly recognized as valuable by-products for their potential uses. For instance, roasted date seeds powder are used to prepare coffee-like beverages, and the seed can also be a potential (edible) oil source especially in arid areas (Farag et al., 2021; Mrabet et al., 2020). Date seed oil (DSO) is abundant in bioactive compounds, such as vitamin E vitamers, phenolic compounds, phytosterols, and others, making it an attractive candidate for functional applications in both the food and non-food sectors (Alkhoori et al., 2022; Echegaray et al., 2021; Farag et al., 2021; Maqsood et al., 2020; Mrabet et al., 2020).

Although edible oils, being recognized to possess lower chemical diversity, compared to other foods, their composition has been linked to terroir. This has been well documented for various edible oils, including extra-virgin olive oil, palm oil, or argan oil, among others (Lucini, Rocchetti, & Trevisan, 2020). Similarly, the chemical composition of DSO is influenced by multiple factors, including date variety and geographical origin, which will affect its biological activities as well as nutritional and functional properties (Abdul-Hamid et al., 2019). Thus, understanding how geographical origin impacts the composition of DSO is critical for quality control, authenticity verification, consumer trust, and economic valorization of date seeds as by-product (Caporale & Monteleone, 2001).

Previous studies mostly focused on major lipid classes of DSO and the influence of date variety, with limited attention to geographical effects (Al Juhaimi et al., 2018; Habib, Kamal, Ibrahim, & Dhaheri, 2013; Harkat et al., 2022; Lieb et al., 2020; Nehdi, Sbihi, Tan, Rashid, & Al-Resayes, 2018). Most sample collections were limited to specific regions, lacking a systematic comparison across different locations. When comparing DSO composition across studies from different countries, noticeable differences emerged, likely due to geographical variation. However, these observations were drawn from separate publications rather than from an individual study, explicitly designed to assess intra-country geographical effects (Al Juhaimi et al., 2018; Habib et al., 2013; Harkat et al., 2022; Lieb et al., 2020; Nehdi et al., 2018).

In a recent study, the main lipid composition of DSO from three Moroccan palm groves (*Allougoum*, *Alnif*, and *Errachidia*) were characterized, aiming at evaluating the effect of geographical location on DSO (El Harkaoui et al., 2024). Conventional lipid analyses, including fatty acids, triacylglycerols (TAG), tocochromanols, and phytosterols, were combined with multivariate statistical approaches such as principal component analysis (PCA) and heatmaps to identify compositional differences. While some trends were observed, differentiation between Moroccan palm groves remained challenging. Nevertheless, the *Allougoum* region exhibited a distinct clustering pattern, associated with variations in minor TAG. Especially minor compounds and patterns thereof provide relevance in authenticity assessment (Qian et al., 2020). These findings suggest that geographical origin may influence DSO composition, even when analyzing major lipid constituents.

While conventional lipid analysis provided valuable insights, it only captured a limited, portion of DSO's chemical diversity, excluding and even neglecting the other important parts of the metabolome, highlighting the need for a broader analytical approach. The previous study was a crucial first step in establishing authenticity markers, but its scope was inherently constrained (El Harkaoui et al., 2024). Moving towards an untargeted metabolomic approach allows for a more comprehensive investigation of DSO composition, potentially identifying novel markers that enhance geographical differentiation. A more wider and comprehensive profile, including the more polar or amphiphilic metabolites, holds significant potential for identifying reliable geographical markers (Hu, Zhang, Xing, Yu, & Chen, 2022).

Obviously, ultra-high-performance liquid chromatography-electrospray ionization quadrupole time-of-flight mass spectrometry (UHPLC-ESI-QTOF-MS) is a well-established technique for untargeted metabolomics, offering high sensitivity and resolution, as well as the capability to detect a diverse range of metabolites, including polar compounds that are often neglected in traditional lipid analysis. This approach has been widely employed in the geographical authentication of edible oils, particularly in olive oil (Ghisoni et al., 2019; Gil-Solsona et al., 2016; Kalogiouri, Aalizadeh, & Thomaidis, 2018; Mohamed et al., 2018; Willenberg, Parma, Bonte, & Matthäus, 2021), where secondary metabolites have been successfully used as markers. Given the effectiveness of metabolomic profiling in other oil types, applying this approach to DSO represents a logical extension of the previous work (El Harkaoui et al., 2024), with the potential to reveal novel compositional markers linked to geographical origin. However, a major challenge of untargeted metabolomics is the vast amount of data generated, necessitating advanced statistical and computational methods for effective interpretation. The high dimensionality of metabolomic datasets requires multivariate statistical techniques such as PCA, orthogonal partial least squares-discriminant analysis (OPLS-DA), and variable importance in projection (VIP) scores to extract meaningful patterns. These chemometric tools enhance classification accuracy by identifying key metabolites responsible for regional compositional differences, thus, improving the discrimination of samples based on origin (Mattoli, Gianni, & Burico, 2022; Yi et al., 2016). However, it remains unclear, if wheter DSO from different Moroccan origins, can be differentiated from each other, and wheter authentication is possible not only for economic reasons, but also with regard to food safety. A strategy with appropriate food analytical methodologies must be developed. This would enhance marketability of Moroccan DSO, but can also serve as another scientific example for food authenticity testing.

The main aim of the present study was to establish, for the first time, a comprehensive untargeted metabolomic profile of Moroccan DSO using UHPLC-ESI-QTOF-MS combined with advanced chemometric analysis, and to evaluate whether this profile varies across different palm groves within Morocco. Specifically, the study investigates compositional differences among samples from *Allougoum*, *Alnif*, and *Errachidia*, and seeks to identify key polar metabolites that may serve as geographical markers. This work builds on previous studies focusing on the analysis of major lipid constituent and contributes new insights into the authentication and traceability of Moroccan DSO, while also highlighting the potential of date seeds as valuable by-products for different functional applications. A high quality of such novel approaches is mandatorily to gain consumer trust.

## 2 Material and methods

### 2.1 Material

**Fig. 1:**
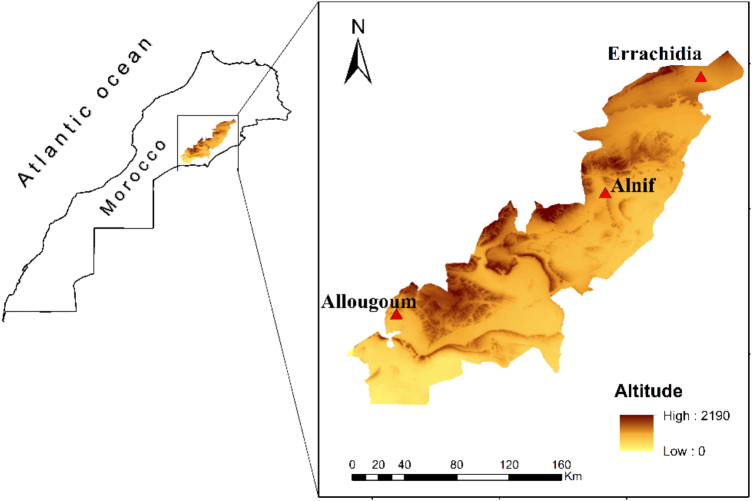
Sampling provenances *Allougoum*, *Alnif*, and *Errachidia* (red triangles) with regard to location and altitude. Figure reproduced from (El Harkaoui et al., 2024), licenced under CC BY 4.0. (For interpretation of the references to color in this figure legend, the reader is referred to the web version of this article.)

The sample set used consists of 26 date seed samples from three Moroccan provenances: *Allougoum* (Ag), *Alnif* (Al), and *Errachidia* (Er). The exact location of the sampling provenances is visualized in Fig. 1. These are the same samples used in a previous publication (El Harkaoui et al., 2024), where detailed information on sample codes, collection sites, and environmental parameters such as average temperature and annual rainfall is provided. The varieties of date seeds included were ‵Amchaw', ‵Berhi', ‵Boufeggous', ‵Bouhmroune', ‵Bousthammi', ‵Ikhlas', ‵Iklan', ‵Jihel', ‵Khalt', (clone) ‵Lmtrwah', ‵Mejhoul', ‵Racetmar', ‵Sayer', ‵Tahmout', and ‵Tarzawa'. DSO was extracted using hexane (Promochem, Picograde quality, LGC Standards GmbH, Wesel, Germany) for 6 h in a Twisselmann apparatus, following the standard methods outlined by the *German Society for Fat Science* (DGF B—I 5 (12) and B-II 4a (09)) (DGF, 2021). Vanillin (99 %), indole-3-carboxaldehyde (97 %), and *n*-(3-oxohexanoyl) homoserine lactone (97 %) (Merck KGaA, Darmstadt, Germany) were used as reference compounds.

### 2.2 Sample preparation

The polar extract from the Moroccan DSO samples was obtained following an optimized method reported by (Willenberg et al., 2021) with some minor modifications. In brief, 1 g DSO was weighed in a 10 mL glass tube and 2 mL of the extraction solvent were added (methanol/H_2_O, 80:20, (*v*/v)). The mixture was shaken vigorously for 1 min at 1500 min^−1^ (VXR basic Vibrax; IKA Werke GmbH & Co. KG, Staufen, Germany), then centrifuged (1550 ×*g*, 15 min) and 1.5 mL of the resulting supernatant were collected into a new 10 mL-glass tube. The remaining lower phase was extracted a second time in the same manner and 2 mL of the second supernatant were combined with the first extract. The combined extracts were evaporated to dryness at 40 °C with 1 mbar vacuum using a rotational vacuum concentrator (RVC 233 CDplus, Martin Christ Gefriertrocknungsanlagen GmbH, Osterode am Harz, Germany). The residue was dissolved in 0.25 mL of methanol/H_2_O (80:20, *v*/v), shaken vigorously for 1 min (1,500 min^−1^) and transferred into a 1.5 mL-microreaction tube. After centrifugation for 5 min at 16,000 ×g, the supernatant was transferred into an HPLC vial with a glass insert. Once the samples were processed as described above and filled into vials, a pooled sample (quality control, QC) was prepared by combining equal volumes from each vial and vigorously mixing them into a homogenous pooled sample.

### 2.3 Instrumentation

The untargeted analysis of the polar extract was carried out using an UHPLC-ESI-QTOF-MS approach. For the UHPLC (Ultimate 3000 Series, Thermo Fisher Scientific Inc., Waltham, MA, USA), samples were kept in the autosampler at 10 °C until injection of a 3 μL aliquot. Separation was carried out at 40 °C on a 1.7 μm × 150 mm × 2.1 mm Kinetex EVO C18-reversed phase column with a SecurityGuard ULTRA sub-2 μm pre-column (Phenomenex Ltd. Deutschland, Aschaffenburg, Germany). An aqueous solution of 0.1 % formic acid (98 % p.a., Honeywell Specialty Chemicals Seelze GmbH, Seelze, Germany) was used as eluent A, and methanol (Honeywell Specialty Chemicals Seelze GmbH, Seelze, Germany) acidified with 0.1 % formic acid was used as eluent B. The elution began with 20 % eluent B and a flow rate of 0.3 mL/min. A gradient was then used, starting with a linear increase from 20 % to 65 % B over 1 to 2.5 min, followed by a linear increase from 65 % to 99 % B over 2.5 to 8 min, and an isocratic hold at 99 % B from 8 to 11 min. Finally, the system was re-equilibrated to the initial conditions (20 % eluent B) in less than a minute, and the system was left for 3 min until the next start.

The mass spectrometric detection was done after electrospray ionization (positive and negative modes) using a QTOF-MS (impact HD, Bruker Daltonics GmbH & Co. KG, Bremen, Germany). The data were acquired over an *m/z* ratio ranging from 50 to 1000 Da using the following TOF parameters: capillary voltage 4500 V, drying gas temperature 250 °C, dry gas flow 10 L/min, nebulizing gas pressure 3 bar and plate offset -500 V. Tune parameter were: Funnel 1 RF 150 Vpp; funnel 2 RF 200 Vpp, isCID energy 0 eV, hexapole RF 100 Vpp, ion energy 4 eV, low mass 50 *m*/*z*, collision RF 500 Vpp, transfer time 100 μs, pre-pulse storage 5 μS. MS/MS parameters: bbCID mode, collision energy MS mode 7 eV, MS/MS mode 40 eV. The Compass 2023b for otof series package with otofControl 6.3 (Bruker Daltonics GmbH & Co. KG, Bremen, Germany) was used for data acquisition. The system was calibrated using sodium formate solution (10 mM) injected at 0.18 μL/min. During the first 30 s of every run. Samples were injected randomly in triplicates, with QC and blank samples analyzed every ten injections to monitor instrument performance.

### 2.4 Data processing

**Fig. 2:**
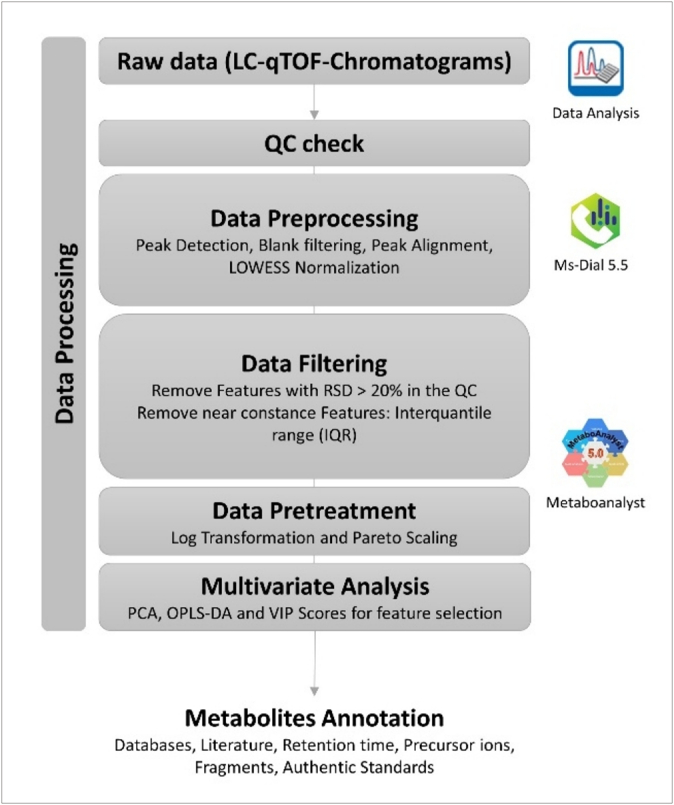
Workflow from data processing to metabolites annotation.

The mass calibration processing and initial inspection was performed using DataAnalysis 6.1 (Bruker Daltonics GmbH & Co. KG, Bremen, Germany). For further data processing (peak picking, retention time alignment, and normalization), the raw data set of the analyzed samples was converted into *.abf files using Analysis Base File Converter (Reifycs Inc., Tokyo, Japan) to make them compatible with the software MS-Dial version 5.5 (RIKEN Center, Kanagawa, Japan). The data collection was done within the retention range 1 min to 12 min and a mass range of 50–1000 Da. For peak detection, a minimum peak height of 5000 (negative mode) and 10,000 (positive mode), and the selected adduct were [M + H]^+^ and [M-H]^−^ for the positive and negative mode, respectively. The QC sample in the middle of the batch was selected as an alignment sample with a retention time tolerance of 0.5 min and a MS tolerance of 0.015 Da. The complete applied MS-Dial parameters, including those used for data collection, peak detection and alignment are reported in Table S1. MS-Dial integrated LOWESS drift normalization was performed on the data set to reduce systematic variations and ensure reproducibility. The resulting feature list which correspond to a distinct compound with the associated *m*/*z* value and retention time was exported to a .csv file (Microsoft, Redmont, WA, USA) for further steps. Following, the data was filtered, log-transformed, and Pareto-scaled before undergoing multivariate analysis in MetaboAnalyst 5.0 (Pang et al., 2021). Subsequently, a selection of potential markers for discriminating the geographical origin of DSO oil was compiled based on the processed data. The overall data processing workflow is summarized in Fig. 2.

### 2.5 Screening and identification

Significant features were selected, excluding those resulting from in-source fragmentation. Molecular formulas were generated using Bruker‘s *Smart Formula* tool in DataAnalysis 6.1, applying a mass deviation threshold of <5 ppm and an isotope distribution deviation of <20 %. These formulas, along with exact masses, were then searched and tentatively annotated using online metabolite databases, including LipidMaps (https://www.lipidmaps.org/, last accessed 22/11/2024), Mass Bank of North America (https://mona.fiehnlab.ucdavis.edu/, last accessed 22/11/2024), and Foodb (https://foodb.ca/, last accessed 22/11/2024). Annotations from different databases were crosschecked, and, when available, MS/MS spectra with acquisition parameters similar to ours were considered for fragment comparison. When applicable, authentic standards were also used to confirm the annotated features. To ensure reliable identification, features were classified based on the confidence level criteria proposed by Schymanski et al. (2014) and Sumner et al. (2007). In the present study, annotation levels were assigned as follows: Level 1 (confirmed structure with an authentic standard), Level 2 (probable structure, single candidate based on comparison with databases MS/MS spectra), Level 3 (tentative candidate(s)), Level 4 (unequivocal molecular formula), and Level 5 (unknown, exact mass).

## 3 Results and discussion

### 3.1 Data elaboration

To obtain comprehensive information on the metabolites present in Moroccan DSO, the polar fraction of the oil samples was extracted and analyzed using UHPLC-ESI-QTOF-MS. This study aimed to characterize the metabolomic profile of DSO for the first time and to compare these findings with previously reported chemical composition data, assessing the effect of geographical origin on the metabolite profile (El Harkaoui et al., 2024). Additionally, marker identification and structure elucidation were key objectives.

**Fig. 3:**
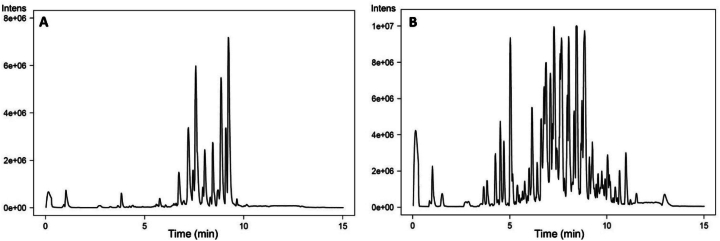
**A&B.** Comparison of the base peak chromatograms (BPCs) of the polar extract derived from date seed oil depending on the ionization mode. (A) negative ion mode and (B) positive ion mode, obtained using UHPLC-ESI-QTOF-MS. These chromatograms are representative of a typical sample and illustrate the higher signal complexity and feature richness observed especially for the positive ionization mode.

Analyses were conducted in both positive and negative electrospray ionization modes to maximize metabolite detection. Overall, more features were observed in the positive mode than in the negative mode, as illustrated in the base peak chromatograms (BPC) of a representative DSO sample (Fig. 3).

The BPCs of ten quality control (QC) samples were overlaid (Fig. S1) and inspected for peak intensity and retention time stability. The high overlap in BPCs across QC samples in both modes indicates the stability of the analyzing method. Following data acquisition, log transformation and Pareto Scaling were applied, and an initial principal component analysis (PCA) was performed. This step ensured the quality of normalization by assessing the clustering of QC samples and the separation of blanks. As expected, QC samples clustered closely with DSO samples, while blanks were distinctly separated (Fig. S2). These findings align with recommended metabolomics guidelines (Broadhurst et al., 2018; Mattoli et al., 2022), confirming the robustness of the analytical method and data pre-processing.

To refine the dataset, statistical data filtering was implemented. Features with a relative standard deviation (RSD) exceeding 20 % in QC samples were removed. Additionally, variables with low variance across experimental conditions were excluded based on interquartile range analysis. Such filtering enhances data quality by eliminating variables unlikely to contribute meaningfully to statistical models (Mattoli et al., 2022). Following data filtration, 446 features remained in the negative mode and 1654 features in the positive mode. The predominance of features in positive mode could be attributed to the ionization efficiency of certain metabolites or more related to the instrumental settings favouring positive ion formation. This trend was consistent with previous metabolomic studies of oils, which frequently reported a higher number of detected features in positive mode (Dou et al., 2025; Gil-Solsona et al., 2016; Hu et al., 2022; Willenberg et al., 2021). The filtered datasets were subsequently subjected to multivariate analysis with log transformation and Pareto scaling, as recommended for metabolomics data (Di Guida et al., 2016; Van den Berg et al., 2006).

### 3.2 Multivariate analysis

**Fig. 4:**
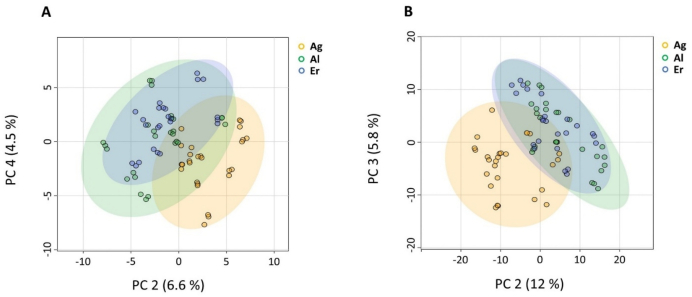
**A&B.** PCA Score plot visualization in negative (A) and positive (B) ionization modes comparing samples from the three palm groves: *Allougoum* (Ag), *Alnif* (Al), and *Errachidia* (Er).

A new PCA was conducted without QC samples to characterize the differentiation among DSO samples from three geographical regions: *Allougoum*, *Alnif*, and *Errachidia* (Fig. 4). As an unsupervised method, PCA enables dimensionality reduction while offering insights into clustering patterns. Overall, the first eight principal components explained over 80 % of total variance in both modes as shown by the scree plots (Fig. S3). PC1 accounted for 65 % of total variance in negative mode and 49 % in positive mode, however, the variance captured by PC1 was not related to geographical origin. The optimal projection for distinguishing the three regions was achieved in the PC2–PC4 plane for negative mode and the PC2–PC3 plane for positive mode (Fig. 4). The PCA score plot revealed no clear separation between samples from *Alnif* and *Errachidia*, which was anticipated given their geographical proximity (Fig. 4) and likely similarities in soil type, environmental conditions, and cultivations practices affecting metabolite composition. However, samples from *Allougoum*, located further south at a lower altitude and closer to the Atlantic Ocean, exhibited a modest clustering trend distinct from the other two locations (Fig. 1 and Fig. 4). This trend was consistent across both ionization modes (Fig. 4) and aligns with previous findings based on chemical composition, where samples from *Allougoum* demonstrated a clustering as compared to the samples from *Alnif* and *Errachidia* (El Harkaoui et al., 2024).

Given this observed trend, orthogonal partial least squares discriminant analysis (OPLS-DA) was applied to identify markers distinguishing *Allougoum* samples from the rest. In this analysis, samples from *Alnif* and *Errachidia* were combined into a single group (AlEr) for comparison. According to established criteria, a valid OPLS-DA model should have Q2 > 0.5, R2Y > 0.7, and a difference between R2Y and Q2 not exceeding 0.2–0.3 (Eriksson et al., 2003). The generated OPLS-DA model exhibited a Q2 value exceeding 0.5 and an R2Y value greater than 0.7, indicating model validity.

**Fig. 5:**
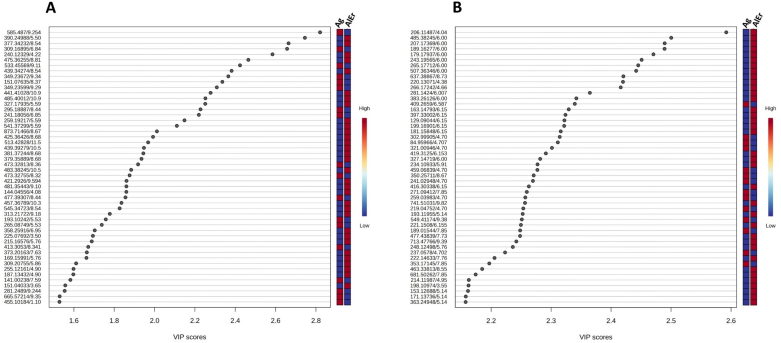
**A&B**. Top 50 discriminating features for geographical origin, ranked by their VIP scores, shown for negative ion mode (A) and positive ion mode (B). Samples from *Allougoum* (Ag) are compared with the combined group of *Alnif* and *Errachidia* (AlEr). The color bars represent the median intensity of each feature across the sample groups. Tables S2 and S3 indicate the same top-ranking features with the corresponding ordering numbers (from 1 to 50) and the exact VIP score for each feature.

**Fig. 6:**
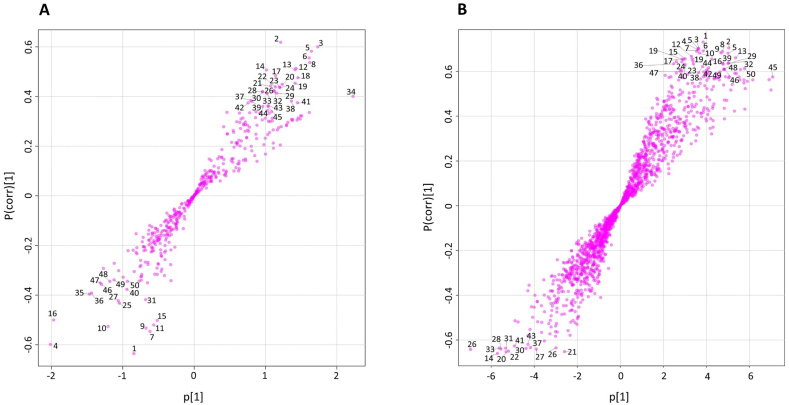
**A&B**. S-plots for negative ion mode (A) and positive ion mode (B). The numbered markers (1 to 50) correspond to the top 50 discriminating features ranked by VIP scores, as reported in Fig. 5 and listed in Tables S2 and S3.

To identify key variables contributing to the classification, variable importance projection (VIP) scores were generated for both positive and negative mode datasets. The VIP score summarizes a variable's contribution to the model and is calculated as a weighted sum of the squared correlations between the OPLS-DA components and the original variables (Mohamed et al., 2018). Features were ranked based on their VIP scores, and only the top 50 were selected for further investigation to attempt their identification (Fig. 5 and Table S2 and S3). In addition to the VIP scores, the S-plot from the OPLS-DA model was also used to visualize the relationship between the covariance and correlation of each variable, aiding in the selection of reliable markers. The top 50 features also appeared as significant contributors in the corresponding S-plot (Fig. 6); their distribution in the top-right and bottom-left quadrants confirmed their strong influence in driving the separation between sample groups.

### 3.3 Compound identification and interpretation

Among the top 50 features, features identified with confidence levels ranging from Level 4 to Level 1 are reported in Table 1, Table 2.

**Table 1:** Tentatively identified markers contributing to the geographical origin discrimination (negative mode).

No.	VIP score	MS (*m*/*z*)	RT (min)	Adducts	Formula	Δ (ppm)	Compound	Class (Subclass)	Annotation confidence
1	2.820	586.49427	9.25	[M-H]^−^	C_38_H_66_O_4_	3.1	octacosyl ferulate	cinnamic acids and derivatives (hydroxycinnamic acids and derivatives)	Level 3
2	2.745	391.25715	5.05	[M-H]^−^	C_19_H_37_NO_7_	−1.0	–	–	Level 4
5	2.585	241.13056	4.22	[M-H]^−^	C_12_H_19_NO_4_	3.5	n-(3-oxohexanoyl) homoserine lactone	carboxylic acids and derivatives (amino acids and derivatives)	Level 1
6	2.465	476.36982	8.82	[M-H]^−^	C_26_H_52_O_7_	−3.1	1-O-α-d-glucopyranosyl-1,2-eicosandiol	fatty acyls (fatty acyls glycosides)	Level 3
8	2.382	440.35001	8.55	[M-H]^−^	C_26_H_48_O_5_	−0.4	momordol	fatty acyls (fatty alcohols)	Level 3
9	2.366	350.24399	9.34	[M-H]^−^	C_16_H_34_N_2_O_6_	5.0	–	–	Level 4
13	2.252	486.40739	10.91	[M-H]^−^	C_32_H_54_O_3_	−0.9	–	–	Level 4
16	2.219	242.18783	6.85	[M-H]^−^	C_14_H_26_O_3_	−1.5	hydroxy tetradecenoic acid	fatty acyls (hydroxy fatty acids)	Level 3
17	2.148	260.19944	5.59	[M-H]^−^	C_14_H_28_O_4_	2.6	dihydroxy tetradecanoic acid	fatty acyls (hydroxy fatty acids)	Level 3
20	1.992	426.37153	8.68	[M-H]^−^	C_26_H_50_O_4_	0.2	–	–	Level 4
21	1.968	514.43555	11.59	[M-H]^−^	C_34_H_58_O_3_	−5.9	–	–	Level 4
25	1.918	474.33540	8.37	[M-H]^−^	C_29_H_46_O_5_	0.7	–	–	Level 4
29	1.860	482.36170	9.10	[M-H]^−^	C_28_H_50_O_6_	2.0	certonardosterol M	steroids and steroid derivatives (bile acids, alcohols and derivatives)	Level 3
30	1.860	145.05283	4.08	[M-H]^−^	C_9_H_7_NO	0.5	indole-3-carboxaldehyde	indoles and derivatives (Indoles)	Level 1
32	1.836	458.37516	10.33	[M-H]^−^	C_30_H_50_O_3_	−3.0	–	–	Level 4
36	1.738	266.09476	5.53	[M-H]^−^	C_17_H_14_O_3_	1.8	7-methoxy-2-methylisoflavone	isoflavonoids (*o*-methylated isoflavonoids)	Level 3
37	1.703	359.26643	6.96	[M-H]^−^	C_19_H_37_NO_5_	−3.0	–	–	Level 4
39	1.689	216.17303	5.77	[M-H]^−^	C_12_H_24_O_3_	2.2	12-hydroxydodecanoic	fatty Acyls (hydroxy fatty acids)	Level 2
47	1.557	152.04760	3.65	[M-H]^−^	C_8_H_8_O_3_	1.68	vanillin	phenols	Level 1

The class of the compounds were attributed via ClassyFire and LipidMaps; No: the order of the compounds in the list of 50 top ranked features reported in Table S2 and Fig. 4. The MS/MS spectra for compounds identified at Level 1 are provided in Figs. S4–S6, and the main MS/MS fragments for compounds identified at Level 2 are summarized in Table S4.

**Table 2:** Tentatively identified markers contributing to the geographical origin discrimination (positive mode).

No.	VIP score	MS (m/z)	RT (min)	Adducts	Formula	Δ (ppm)	Compounds	Class (Subclass)	Annotation confidence
2	2.500	484.37499	6.00	[M + H]^+^	C_28_H_52_O_6_	−4.0	–	–	Level 4
3	2.489	206.16689	6.00	[M + H]^+^	C_14_H_22_O	−3.5	–	–	Level 4
16	2.324	396.32427	6.15	[M + H]^+^	C_24_H_44_O_4_	0.8	dihydroxy tetracosadienoic acid	fatty Acyls (hydroxy fatty acids)	Level 3
18	2.322	198.16224	6.15	[M + H]^+^	C_12_H_22_O_2_	−1.3	δ-dodecalactone	lactones (delta valerolactones)	Level 2
19	2.316	180.1513	6.15	[M + H]^+^	C_12_H_20_O	0.6	2,4-dodecadienal	fatty acyls (fatty aldehydes)	Level 2
25	2.277	233.10307	5.91	[M + H]^+^	C_10_H_17_O_6_	0.0	–	–	Level 4

The class of the compounds were attributed via ClassyFire and LipidMaps; No: the order of the compounds in the list of 50 top ranked features reported in Table S3 and Fig. 4. The MS/MS spectra for compounds identified at Level 1 are provided in Figs. S4–S6, and the main MS/MS fragments for compounds identified at Level 2 are summarized in Table S4.

**Fig. 7:**
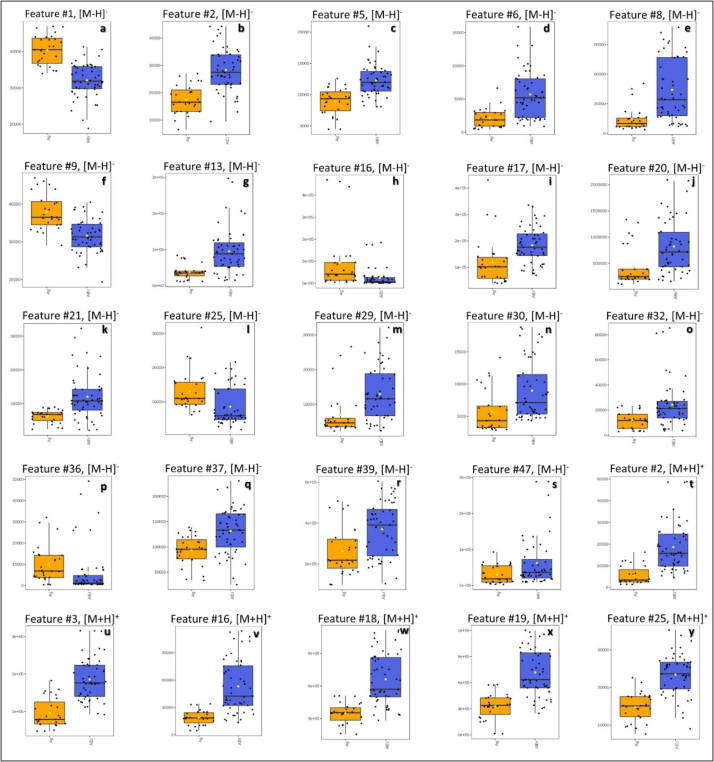
a-y. Variation of the 25 tentatively identified metabolites (listed in Table 1, Table 2) detected in both negative and positive ionization modes. These 25 features are part of the top 50 most significant features (ranked 1–50) based on their VIP scores. Each subfigure is labeled with the corresponding feature number (feature #number), matching the markers in Table 1, Table 2, and also indicates the ionization mode: negative ([M + H]^−^) or positive ([M + H]^+^). Samples from *Allougoum* (Ag, orange) are compared with the combined *Alnif* and *Errachidia* group (AlEr, blue).

The distribution of the tentatively identified metabolites is visualized in box plots (Fig. 7), showing their presence across all DSO samples. This suggests that differentiation between geographical origins is driven more by concentration differences than by the presence or absence of specific metabolites, which was expected, as all samples belong to the same type of oil (DSO). The concentration of individual metabolites varied, with some being higher in *Allougoum* and others in the combined *Alnif*-*Errachidia* group. Although precise discrimination thresholds for practical authenticity testing cannot be defined yet, the observed differences in relative intensities across the regions, as illustrated by box plots, suggest preliminary ranges that could guide future targeted investigations.

The box plots and PCA plots (Fig. 4, Fig. 7) also revealed a wide dispersion of samples from the same provenance within the cluster. As all samples were collected at the same maturity stage (*Tamr* stage) and processed under identical conditions, including oil extraction, and storage, this intra-regional variability may also reflect the genetic background of the date varieties included in the sampling. A similar pattern was also reported in a previous publication (El Harkaoui et al., 2024). However, disentangling these effects requires a larger dataset with a broader representation of varieties within each region to allow robust statistical evaluation. It should be noted that in Morocco, literature reports more than 200 date varieties, which highlights the challenge of fully assessing varietal effects (Bouhlali, Ramchoun, et al., 2017).

Among the markers, the compound with the highest VIP score was octacosyl ferulate, a hydroxycinnamic acid derivative. This compound has not been previously reported in oils, but its chemical structure suggests that it may result from the esterification of ferulic acid, previously detected in DSO (Hamza et al., 2021; Harkat et al., 2022), with octacosanol, a policosanol commonly found in vegetable oils (Jung et al., 2011). The presence of related ferulate-based compounds, such as γ-oryzanol in vegetable oils (Cuevas et al., 2017), further supports this tentative structure suggestion. The variation of octacosyl ferulate across geographical origins could be influenced by the concentrations of both ferulic acid and octacosanol. However, confirming this compound's identity requires analysis using an authentic standard under identical experimental conditions. Additionally, n-(3-oxohexanoyl) homoserine lactone, indole-3-carboxaldehyde, and vanillin were confirmed by a comparison with authentic standards, with their extracted ion chromatograms (EICs) and corresponding MS/MS spectra presented in Fig. S4-S6. Indole-3-carboxaldehyde, commonly found in cruciferous vegetables (Palladino et al., 2024), was tentatively identified but not confirmed in corn oil, (Alberdi-Cedeño et al., 2017). Vanillin, another key marker, was previously identified as the second most abundant phenolic compound in Algerian DSO, with variations depending on variety (Harkat et al., 2022). Phenolic compounds are well-known markers for geographical origin, as demonstrated in studies on olive oils (Ghisoni et al., 2019; Kalogiouri et al., 2018; Mohamed et al., 2018; Olmo-García et al., 2019). In the present study, vanillin was the only phenolic compound among the top 50 features.

Furthermore, hydroxy fatty acids, a subclass of fatty acyls, were identified as geographical markers for DSO. Compounds such as hydroxy tetradecenoic acid, dihydroxy tetradecanoic acid, 12-hydroxydodecanoic acid, and dihydroxy tetracosadienoic acid were among the most prominent markers. A metabolomic study on Spanish extra virgin olive oil tentatively identified 9,13-dihydroxy-11-octadecenoic acid as one of the significant markers for origin discrimination (Gil-Solsona et al., 2016), further underscoring the potential of hydroxy fatty acids as geographical markers. Additionally relevance of hydroxy fatty acids in general as authenticity markers has been also emphasized in a recent study (Koch et al., 2022). Hydroxy fatty acids are widespread in plant genera such as *Apocynaceae*, *Asteraceae*, *Brassicaceae*, *Coriariaceae*, *Euphorbiaceae*, *Fabaceae*, *Malpighiaceae*, and *Papaveraceae*. The biosynthesis of hydroxy fatty acids can be attributed to desaturases enzymes, which, beyond desaturation, can also catalyze hydroxylation due to mutations that alter substrate interactions (Cahoon & Li-Beisson, 2020). For example, a variant of the FAD2 enzyme converts oleic acid (D9–18:1) into ricinoleic acid (12-OH-D9–18:1), the main component of castor oil, by adding a hydroxyl (-OH) group instead of a double bond forming linoleic fatty acid (Schmid, 2021). In the present study, a C24 dihydroxy fatty acid (dihydroxy tetracosadienoic acid) was tentatively identified, whose elemental formula corresponds to wuhanic acid. However, due to the absence of an authentic standard and reference MS/MS spectra in the literature, it was assigned a Level 3 identification. Evidence supporting the biosynthesis of wuhanic acids comes from studies involving the transgenic expression of candidate biosynthetic enzymes in *Arabidopsis* spp. These enzymes were identified from the transcriptome of developing seeds of Chinese violet cress (*Orychophragmus violaceus*). The findings revealed that the C-18 hydroxyl group is introduced by a FAD2-type hydroxylase, while the C-7 hydroxyl group originates from a divergent FAE1-encoded 3-ketoacyl-CoA synthase (KCS), operating through a unique biosynthetic mechanism referred to as “discontinuous elongation” (Li et al., 2018). This may suggest that fatty acids and hydroxy fatty acids may share common enzymatic pathways. Following this hypothesis, the hydroxy fatty acids identified in the present study correspond to C12, C14, and C24, which aligns, in terms of carbon chain length, with fatty acids identified in a previous study (El Harkaoui et al., 2024). Specifically, lauric acid (C12:0), myristic acid (C14:0), and lignoceric acid (C24:0) were previously reported, suggesting that the hydroxy fatty acids may originate enzymatically, as discussed earlier. Environmental factors influencing enzyme activity in fatty acid synthesis have been well documented (Ghaffari et al., 2023; Porokhovinova et al., 2022; Sidorov & Tsydendambaev, 2014), and such factors may also affect the variation of hydroxy fatty acids. For example, fluctuations in ricinoleic acid in castor oil have been observed in response to irrigation and water deficit (Ramanjaneyulu et al., 2013), supporting the hypothesis that the variation of hydroxy fatty acids in DSO could be influenced by environmental conditions.

Interpreting the chemical metabolites responsible for the geographical variation of DSO remains challenging, especially as the present study is the first to investigate its metabolomic profile. While fats and oils are generally less influenced by geographical origin compared to other foods, impact of the origin has been demonstrated in various types of oil (Lucini et al., 2020). However, in the context of untargeted metabolomics, the relationship between geographical location, defined ecophysiological factors, and the resulting metabolite profile is rarely discussed in detail, making comparative analysis difficult. This challenge is even more pronounced for less-studied oils like DSO, where data is still limited.

On the other hand, previous metabolomic studies already characterized date palm fruits, seeds, and pollen, but they did not specifically address the influence of geographical origin. When comparing the molecular formula reported in these studies, it was found that compounds with the formulas C_18_H_30_O_3_ (tentatively identified as hydroxyoctadecatrienoic acid), C_7_H_6_O_4_ (tentatively identified as gentisic acid), C10H10O4 (tentatively identified as ferulic acid), and C_9_H_10_O_4_ (tentatively identified as homovanillic acid) occurred also in the present dataset (Alsuhaymi et al., 2023; Harkat et al., 2022; M AbouZeid et al., 2022). However, these compounds did not rank among the top 50 features in the present analysis (according to the VIP scores), implying that they are not relevant for the classification of a geographical origin for these samples.

## 4 Conclusion

The present study investigated the effect of geographical location on the metabolomic profile of Moroccan date seed oil (DSO) using an untargeted approach with UHPLC-QTOF-MS. Samples from three Moroccan palm groves (*Allougoum*, *Alnif*, and *Errachidia*) were analyzed, revealing a modest clustering trend linked to geographical origin. These results support the potential of untargeted metabolomics, combined with chemometric modelling, as a powerful tool for origin verification of DSO. Among the top 50 discriminating features identified, 25 metabolites from various chemical classes were tentatively annotated at different confidence levels. Notably, these compounds are reported for the first time in DSO, expanding current knowledge of its chemical complexity. All identified metabolites were present across all samples, indicating that origin-related differentiation is primarily driven by differences in concentration rather than the presence or absence of unique metabolites. The ability to distinguish DSO based on geographical origin adds value to this edible, unconventional and maybe highly valuable oil and supports, as a perspective, the development of region-specific labels. This authenticity aspect will become even more important as DSO continues to gain attention as a source of (edible) oil in arid regions, especially when certain attributes are more strongly associated with oils from specific areas. On the other hand, although the most pronounced variation was observed in samples from *Allougoum*, it should be acknowledged that the overall differences between regions were moderate. This may reflect a relatively stable compositional profile of Moroccan DSO across the studied palm groves. However, further research is needed since this study included only samples from a single crop year and within one country. Future studies should include multi-year sampling and comparisons with DSO from other major date-producing countries, such as Saudi Arabia and the UAE, to better understand how geographical origin influences DSO composition and to validate this initial, promising approach.

## CRediT authorship contribution statement

**Said El Harkaoui:** Writing – original draft, Visualization, Project administration, Investigation, Formal analysis, Conceptualization. **Katharina N'Diaye:** Writing – review & editing, Methodology. **Zoubida Charrouf:** Writing – review & editing, Supervision, Conceptualization. **Sascha Rohn:** Writing – review & editing, Validation, Supervision. **Stephan Drusch:** Writing – review & editing, Validation, Supervision. **Bertrand Matthäus:** Writing – review & editing, Supervision, Funding acquisition.

## Declaration of competing interest

The authors declare that they have no known competing financial interests or personal relationships that could have appeared to influence the work reported in this paper.

## Data Availability

Data will be made available on request.

## Appendix A Supplementary data

Supplementary material contains Fig S1 to Fig S6 and from Table S1 to Table S4. Image 1

## References

[bb0005] Abdul-Hamid N.A., Abas F., Ismail I.S., Tham C.L., Maulidiani M., Mediani A., Zolkeflee N.K.Z. (2019). Metabolites and biological activities of *Phoenix dactylifera* L. pulp and seeds: A comparative MS and NMR based metabolomics approach. Phytochemistry Letters.

[bb0010] AbouZeid M., Afifi A.E.H., Salama A., Hussein R., Youssef F., El-Ahmady S.H., Mohamed Ammar N. (2022). Comprehensive metabolite profiling of Phoenix rupicola pulp and seeds using UPLC-ESI-MS/MS and evaluation of their estrogenic activity in ovariectomized rat model. Food Research International (Ottawa, Ont.).

[bb0015] Al Juhaimi F., Özcan M.M., Adiamo O.Q., Alsawmahi O.N., Ghafoor K., [K.], & Babiker, E. E. (2018). Effect of date varieties on physico-chemical properties, fatty acid composition, tocopherol contents, and phenolic compounds of some date seed and oils. Journal of Food Processing and Preservation.

[bb0020] Alberdi-Cedeño J., Ibargoitia M.L., Guillén M.D. (2017). Bioactive compounds detected for the first time in corn oil: Cyclic dipeptides and other nitrogenated compounds. Journal of Food Composition and Analysis.

[bb0025] Alkhoori M.A., Kong A.S.-Y., Aljaafari M.N., Abushelaibi A., Erin Lim S.-H., Cheng W.-H., Lai K.-S. (2022). Biochemical composition and biological activities of date palm (*Phoenix dactylifera* L.) seeds: A review. Biomolecules.

[bb0030] Alsuhaymi S., Singh U., Al-Younis I., Kharbatia N.M., Haneef A., Chandra K., Jaremko M. (2023). Untargeted metabolomics analysis of four date palm (*Phoenix dactylifera* L.) cultivars using MS and NMR. Natural Products and Bioprospecting.

[bb0035] Bouhlali E.D.T., Alem C., Ennassir J., Benlyas M., Mbark A.N., Zegzouti Y.F. (2017). Phytochemical compositions and antioxidant capacity of three date (*Phoenix dactylifera* L.) seeds varieties grown in the South East Morocco. *Journal of the Saudi Society of*. Agricultural Sciences.

[bb0040] Bouhlali E.D.T., Ramchoun M., Alem C., Ghafoor K., Kashif, Ennassir J., Zegzouti Y.F. (2017). Functional composition and antioxidant activities of eight Moroccan date fruit varieties (*Phoenix dactylifera* L.). Journal of the Saudi Society of Agricultural Sciences.

[bb0045] Broadhurst D., Goodacre R., Reinke S.N., Kuligowski J., Wilson I.D., Lewis M.R., Dunn W.B. (2018). Guidelines and considerations for the use of system suitability and quality control samples in mass spectrometry assays applied in untargeted clinical metabolomic studies. Metabolomics.

[bb0050] Cahoon E.B., Li-Beisson Y. (2020). Plant unusual fatty acids: Learning from the less common. Current Opinion in Plant Biology.

[bb0055] Caporale G., Monteleone E. (2001). Effect of expectations induced by information on origin and its guarantee on the acceptability of a traditional food: olive oil. Sciences des Aliments.

[bb0060] Cuevas M.S., Souza P.T., Da Costa Rodrigues C.E., Meirelles A.J.A. (2017). Quantification and Determination of Composition of Steryl Ferulates in Refined Rice Bran Oils Using an UPLC-MS Method. Journal of the American Oil Chemists’ Society.

[bb0065] DGF (2021).

[bb0070] Di Guida R., Engel J., Allwood J.W., Weber R.J.M., Jones M.R., Sommer U., Dunn W.B. (2016). Non-targeted UHPLC-MS metabolomic data processing methods: A comparative investigation of normalisation, missing value imputation, transformation and scaling. Metabolomics.

[bb0075] Dou X., N’Diaye K., El Harkaoui S., Willenberg I., Ma F., Zhang L., Matthäus B. (2025). Authentication of Virgin Olive Oil Based on Untargeted Metabolomics and Chemical Markers. European Journal of Lipid Science and Technology.

[bb0080] Echegaray N., Gullón B., Pateiro M., Amarowicz R., Misihairabgwi J.M., Lorenzo J.M. (2021). Date Fruit and Its By-products as Promising Source of Bioactive Components: A Review. Food Reviews International.

[bb0085] El Harkaoui S., N'Diaye K., Gharby S., Al-Hilal M., Charrouf Z., Rohn S., Matthäus B. (2024). Insights into date seed oil composition: Geographical variability and potential applications. European Journal of Lipid Science and Technology.

[bb0090] Eriksson L., Jaworska J., Worth A.P., Cronin M.T.D., McDowell R.M., Gramatica P. (2003). Methods for reliability and uncertainty assessment and for applicability evaluations of classification- and regression-based QSARs. Environmental Health Perspectives.

[bb0095] Farag M.A., Otify A., Baky M.H. (2021). *Phoenix Dactylifera* L. Date Fruit By-products Outgoing and Potential Novel Trends of Phytochemical, Nutritive and Medicinal Merits. Food Reviews International.

[bb0100] Ghaffari M., Gholizadeh A., Rauf S., Shariati F. (2023). Drought-stress induced changes of fatty acid composition affecting sunflower grain yield and oil quality. Food Science & Nutrition.

[bb0105] Ghisoni S., Lucini L., Angilletta F., Rocchetti G., Farinelli D., Tombesi S., Trevisan M. (2019). Discrimination of extra-virgin-olive oils from different cultivars and geographical origins by untargeted metabolomics. Food Research International (Ottawa, *Ont.)*.

[bb0110] Gil-Solsona R., Raro M., Sales C., Lacalle L., Díaz R., Ibáñez M., Hernández F.J. (2016). Metabolomic approach for Extra virgin olive oil origin discrimination making use of ultra-high performance liquid chromatography – Quadrupole time-of-flight mass spectrometry. Food Control.

[bb0115] Habib H.M., Kamal H., Ibrahim W.H., Dhaheri A.S.A. (2013). Carotenoids, fat soluble vitamins and fatty acid profiles of 18 varieties of date seed oil. Industrial Crops and Products.

[bb0120] Hamza H., Elfalleh W., Nagaz K. (2021). Date Palm Seed Oil (*Phoenix dactylifera* L.) Green Extraction: Physicochemical Properties, Antioxidant Activities, and Phenolic and Fatty Acid Profiles. Journal of Food Quality.

[bb0125] Harkat H., Bousba R., Benincasa C., Atrouz K., Gültekin-Özgüven M., Altuntaş Ü., Özçelik B. (2022). Assessment of biochemical composition and antioxidant properties of algerian date palm (*Phoenix dactylifera* L.) Seed Oil. Plants.

[bb0130] Hu Q., Zhang J., Xing R., Yu N., Chen Y. (2022). Integration of lipidomics and metabolomics for the authentication of camellia oil by ultra-performance liquid chromatography quadrupole time-of-flight mass spectrometry coupled with chemometrics. Food Chemistry.

[bb0135] Jung D.M., Lee M.J., Yoon S.H., Jung M.Y. (2011). A gas chromatography-tandem quadrupole mass spectrometric analysis of policosanols in commercial vegetable oils. Journal of Food Science.

[bb0140] Kalogiouri N.P., Aalizadeh R., Thomaidis N.S. (2018). Application of an advanced and wide scope non-target screening workflow with LC-ESI-QTOF-MS and chemometrics for the classification of the Greek olive oil varieties. Food Chemistry.

[bb0145] Koch E., Wiebel M., Löwen A., Willenberg I., Schebb N.H. (2022). Characterization of the Oxylipin Pattern and Other Fatty Acid Oxidation Products in Freshly Pressed and Stored Plant Oils. Journal of Agricultural and Food Chemistry.

[bb0150] Li X., Teitgen A.M., Shirani A., Ling J., Busta L., Cahoon R.E., Cahoon E.B. (2018). Discontinuous fatty acid elongation yields hydroxylated seed oil with improved function. Nature Plants.

[bb0155] Lieb V.M., Kleiber C., Metwali E.M., Kadasa N.M., Almaghrabi O.A., Steingass C.B., Carle R. (2020). Fatty acids and triacylglycerols in the seed oils of Saudi Arabian date (*Phoenix dactylifera* L.) palms. International Journal of Food Science & Technology.

[bb0160] Lucini L., Rocchetti G., Trevisan M. (2020). Extending the concept of terroir from grapes to other agricultural commodities: an overview. Current Opinion in Food Science.

[bb0165] Maqsood S., Adiamo O., Ahmad M., Mudgil P. (2020). Bioactive compounds from date fruit and seed as potential nutraceutical and functional food ingredients. Food Chemistry.

[bb0170] Mattoli L., Gianni M., Burico M. (2022). Mass spectrometry-based metabolomic analysis as a tool for quality control of natural complex products. Mass Spectrometry Reviews.

[bb0175] Mohamed M.B., Rocchetti G., Montesano D., Ali S.B., Guasmi F., Grati-Kamoun N., Lucini L. (2018). Discrimination of Tunisian and Italian extra-virgin olive oils according to their phenolic and sterolic fingerprints. Food Research International (Ottawa, Ont.).

[bb0180] Mrabet A., Jiménez-Araujo A., Guillén-Bejarano R., Rodríguez-Arcos R., Sindic M. (2020). Date seeds: A promising source of oil with functional properties. Foods.

[bb0185] Nehdi I.A., Sbihi H.M., Tan C.P., Rashid U., Al-Resayes S.I. (2018). Chemical composition of date palm (*Phoenix dactylifera* L.) seed oil from six saudi arabian cultivars. Journal of Food Science.

[bb0190] Olmo-García L., Wendt K., Kessler N., Bajoub A., Fernández-Gutiérrez A., Baessmann C., Carrasco-Pancorbo A. (2019). Exploring the capability of LC-MS and GC-MS multi-class methods to discriminate virgin olive oils from different geographical indications and to identify potential Origin markers. European Journal of Lipid Science and Technology.

[bb0195] Palladino P., Attanasio L., Scarano S., Degano I., Minunni M. (2024). Colorimetric determination of indole-3-carbaldehyde by reaction with carbidopa and formation of aldazine in ethanolic extract of cabbage. Food Chemistry Advances.

[bb0200] Pang Z., Chong J., Zhou G., Lima Morais D.A., Chang L., Barrette M., Xia J. (2021). Metaboanalyst 5.0: Narrowing the gap between raw spectra and functional insights. Nucleic Acids Research.

[bb0205] Porokhovinova E.A., Matveeva T.V., Khafizova G.V., Bemova V.D., Doubovskaya A.G., Kishlyan N.V., Gavrilova V.A. (2022). Fatty acid composition of oil crops: Genetics and genetic engineering. Genetic Resources and Crop Evolution.

[bb0210] Qian Y., Rudzińska M., Grygier A., Przybylski R. (2020). Determination of triacylglycerols by HTGC-FID as a sensitive tool for the identification of rapeseed and olive oil adulteration. Molecules.

[bb0215] Ramanjaneyulu A.V., Reddy A.V., Madhavi A. (2013). The impact of sowing date and irrigation regime on castor (*Ricinus communis* L.) seed yield, oil quality characteristics and fatty acid composition during post rainy season in South India. Industrial Crops and Products.

[bb0220] Schmid K.M., Ridgway N.D., McLeod R.S. (2021). Biochemistry of Lipids, Lipoproteins and Membranes.

[bb0225] Schymanski E.L., Jeon J., Gulde R., Fenner K., Ruff M., Singer H.P., Hollender J. (2014). Identifying small molecules via high resolution mass spectrometry: Communicating confidence. Environmental Science & Technology.

[bb0230] Sidorov R.A., Tsydendambaev V.D. (2014). Biosynthesis of fatty oils in higher plants. Russian Journal of Plant Physiology.

[bb0235] Sumner L.W., Amberg A., Barrett D., Beale M.H., Beger R., Daykin C.A., Viant M.R. (2007). Proposed minimum reporting standards for chemical analysis chemical analysis working group (CAWG) metabolomics standards initiative (MSI). Metabolomics.

[bb0240] Van den Berg R.A., Hoefsloot H.C.J., Westerhuis J.A., Smilde A.K., van der Werf M.J. (2006). Centering, scaling, and transformations: Improving the biological information content of metabolomics data. BMC Genomics.

[bb0245] Willenberg I., Parma A., Bonte A., Matthäus B. (2021). Development of chemometric models based on a LC-qToF-MS approach to verify the geographic origin of virgin olive oil. Foods.

[bb0250] Yi L., Dong N., Yun Y., Deng B., Ren D., Liu S., Liang Y. (2016). Chemometric methods in data processing of mass spectrometry-based metabolomics: A review. Analytica Chimica Acta.

