# Supplementary material for: Untargeted metabolomic analysis of date seed oil (Phoenix dactylifera L.) using UHPLC-ESI-QTOF-MS - evaluation of the geographical origin effect
Source: Food Chem X. 2025 Oct 14;31:103162. doi: 10.1016/j.fochx.2025.103162 (PMC12640075; doi:10.1016/j.fochx.2025.103162)
Supplement: Supplementary file 1 — Supplementary material contains Fig S1 to Fig S6 and from Table S1 to Table S4. [file mmc1.docx]

**
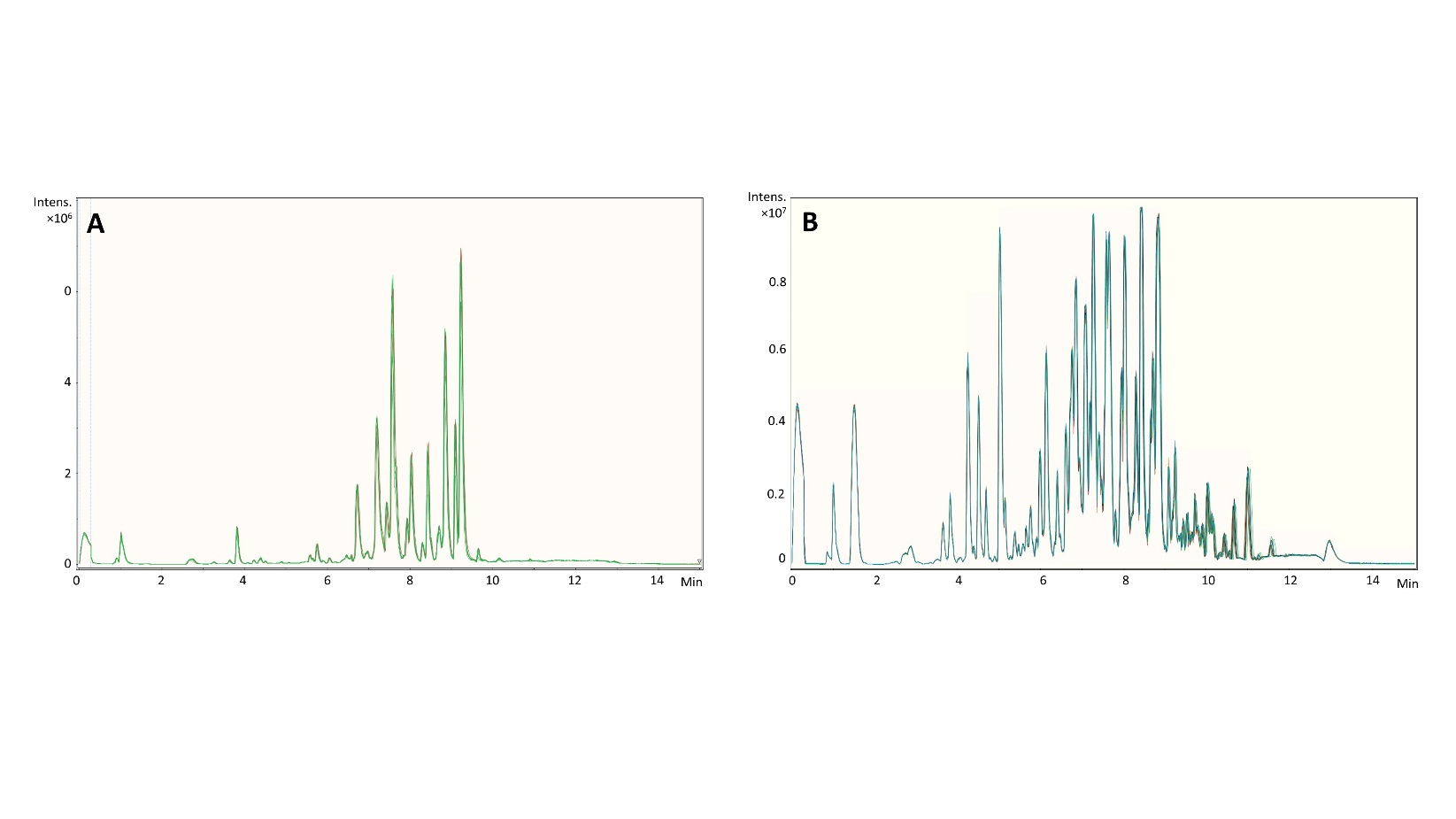
Fig.S1.** Data quality assessment by an overlapped base peak chromatogram of the QC samples in the negative mode (A) and positive mode (B)


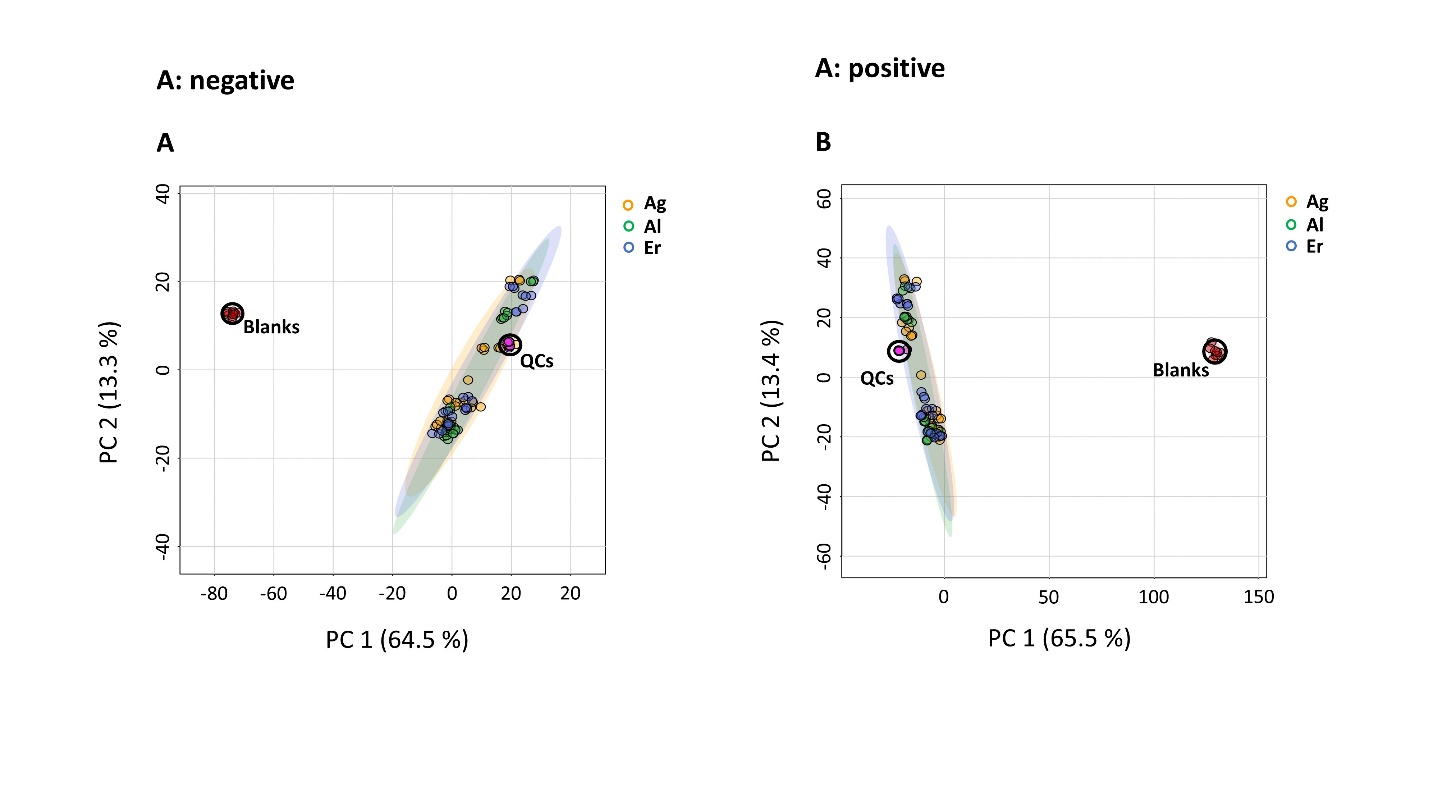


**Fig. S2.** Visual inspection of the score plots including the samples, QCs, and blanks visualization in negative (A) and positive (B) ionization modes

*
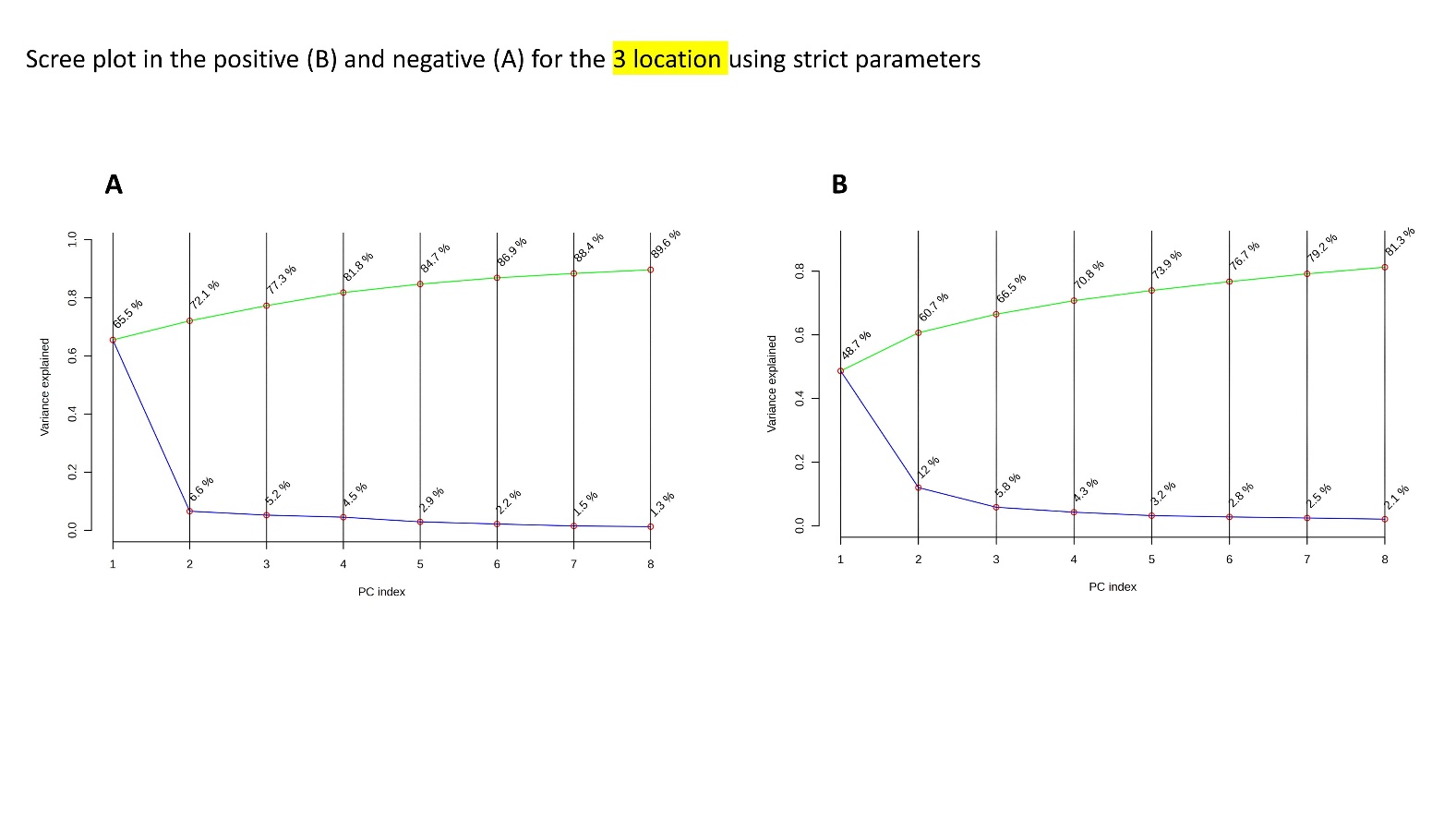
***Fig. S3.** Scree plot visualization in negative (A) and positive (B) ionization modes

**
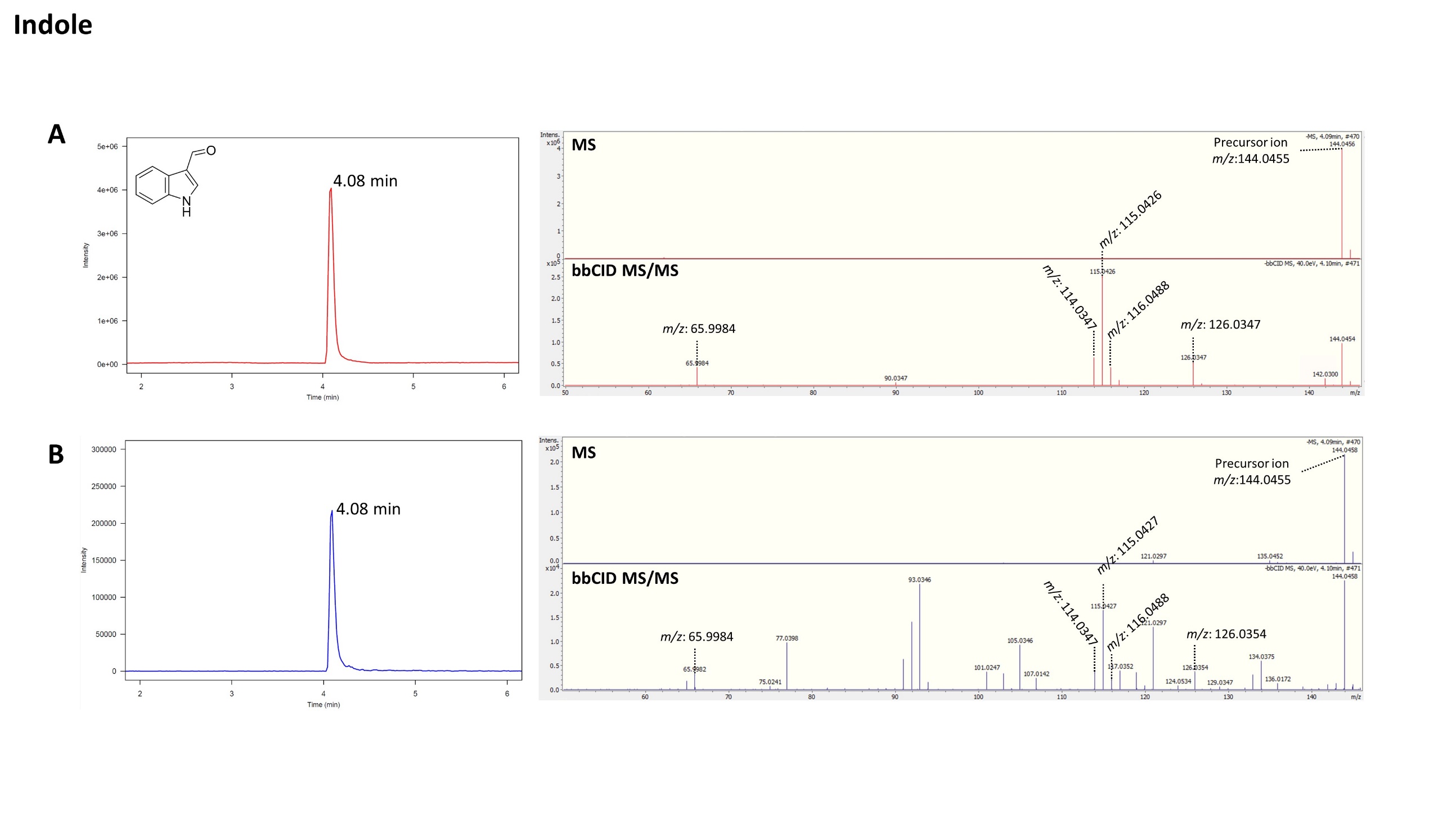
Fig. S4.** Extracted ion chromatogram (EIC) and MS/MS spectrum of indole-3-carboxaldehyde, including annotated fragment ions. (A) Authentic standard; (B) Corresponding signal detected in the DSO sample.


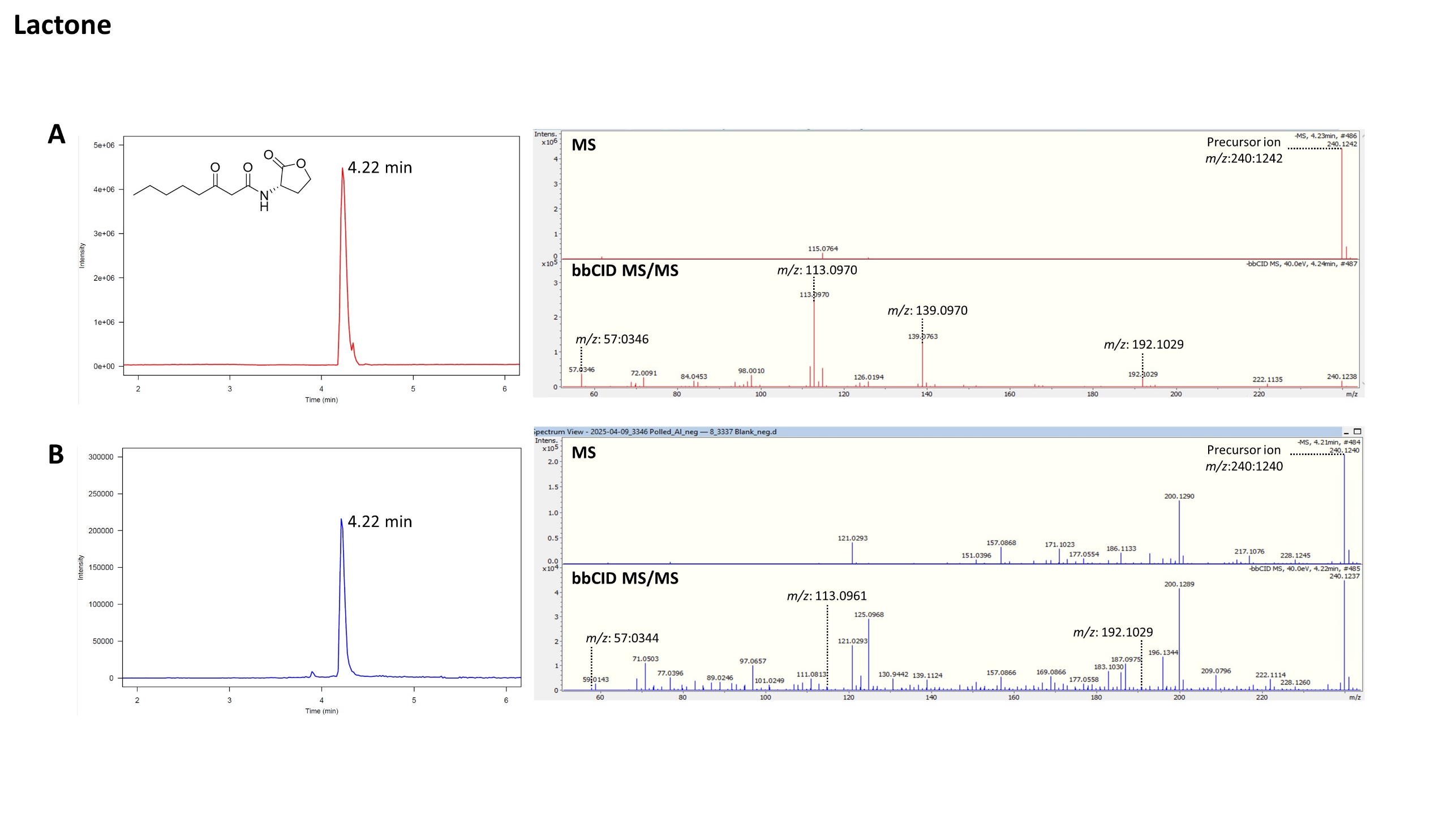
**Fig. S5.** Extracted ion chromatogram (EIC) and MS/MS spectrum of n-(3-oxohexanoyl) homoserine lactone, including annotated fragment ions. (A) Authentic standard; (B) Corresponding signal detected in the DSO sample.


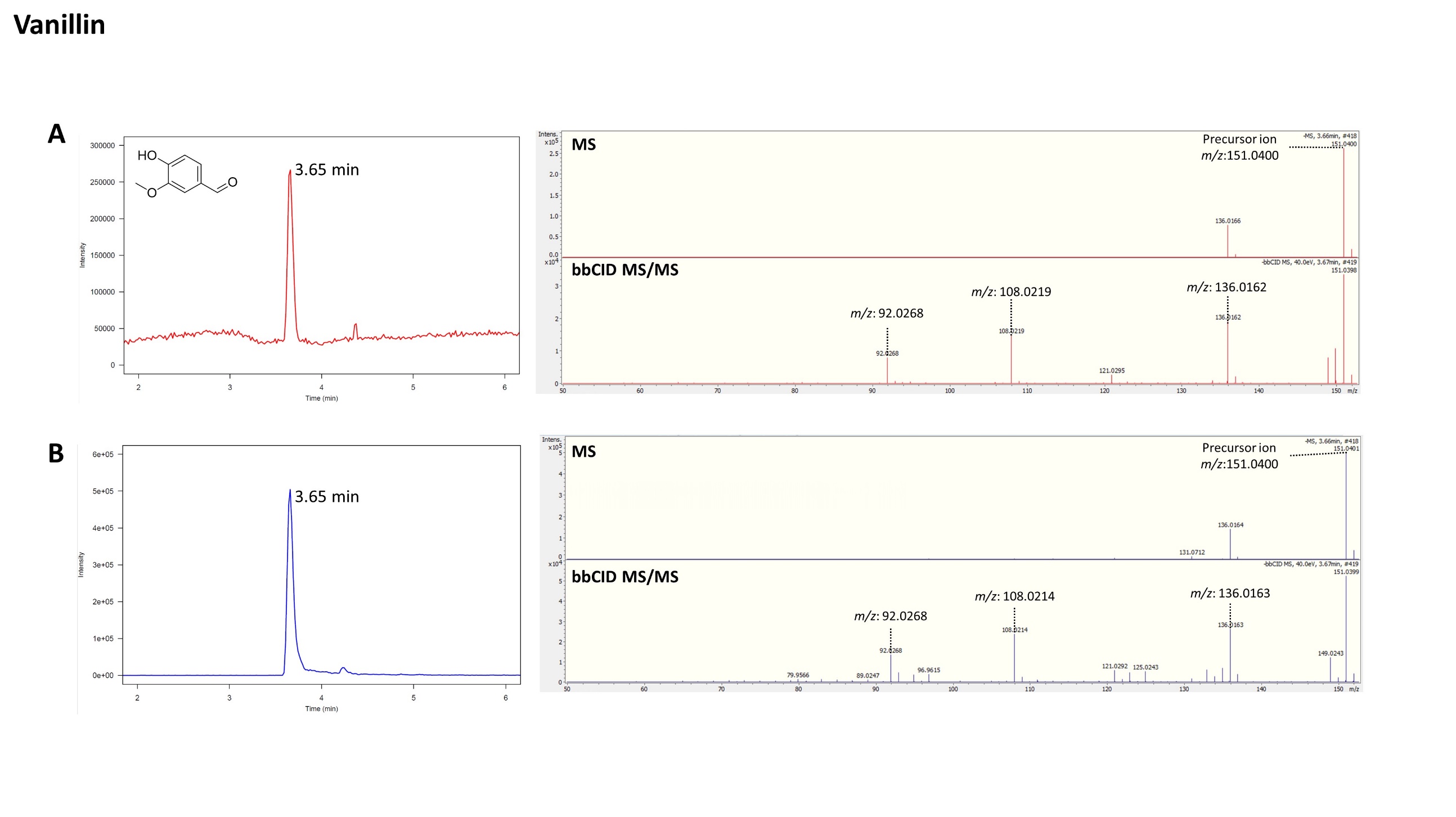
**Fig. S6.** Extracted ion chromatogram (EIC) and MS/MS spectrum of vanillin, including annotated fragment ions. (A) Authentic standard; (B) Corresponding signal detected in the DSO sample.

**Table S1.** Applied MS-Dial (5.5) pre-processing parameters.

| **Data collection** |  |
| --- | --- |
| MS1 tolerance | 0.01 Da |
| Retention time begin | 1 min |
| Retention time end | 12 min |
| MS1 mass range begin | 50 Da |
| MS1 mass range end | 1,000 Da |
| MS/MS mass range begin | 50 Da |
| MS/MS mass range end | 1,000 Da |
| **Peak detection** |  |
| Minimum peak height | 5,000 and 10,000 amplitude for negative and positive modes, respectively |
| Mass slice width | 0.1 Da |
| Smoothing method | Linear weighted moving average |
| Smoothing level | 3 scans |
| **Adduct** |  |
| Positive | [M+H]^+^ |
| Negative | [M-H]^-^ |
| **Alignment** |  |
| Retention time tolerance | 0.05 min |
| MS1 tolerance | 0.015 Da |
| Retention time factor | 50% |
| MS1 factor | 50% |
| Remove features based on blank information | Yes |

**Table S2.** Top 50 features in negative modes classified according to their VIP scores

| **No.** | **Features (*mz*: [M-H]^-^/RT)** | **VIP scores** |
| --- | --- | --- |
| 1 | 585.487/9.254 | 2.820 |
| 2 | 390.24988/5.505 | 2.745 |
| 3 | 377.34232/8.546 | 2.664 |
| 4 | 309.16895/6.846 | 2.657 |
| 5 | 240.12329/4.222 | 2.585 |
| 6 | 475.36255/8.818 | 2.465 |
| 7 | 533.45569/9.114 | 2.424 |
| 8 | 439.34274/8.545 | 2.382 |
| 9 | 349.23672/9.342 | 2.366 |
| 10 | 151.07635/8.372 | 2.336 |
| 11 | 349.23599/9.293 | 2.308 |
| 12 | 441.41028/10.903 | 2.278 |
| 13 | 485.40012/10.913 | 2.252 |
| 14 | 327.17935/5.596 | 2.251 |
| 15 | 295.18887/8.447 | 2.228 |
| 16 | 241.18056/6.854 | 2.219 |
| 17 | 259.19217/5.597 | 2.148 |
| 18 | 541.37299/5.598 | 2.110 |
| 19 | 873.71466/8.676 | 2.012 |
| 20 | 425.36426/8.683 | 1.992 |
| 21 | 513.42828/11.593 | 1.968 |
| 22 | 439.39279/10.563 | 1.947 |
| 23 | 381.37244/8.686 | 1.944 |
| 24 | 379.35889/8.686 | 1.935 |
| 25 | 473.32813/8.366 | 1.918 |
| 26 | 483.38245/10.563 | 1.884 |
| 27 | 473.32755/8.329 | 1.876 |
| 28 | 421.2926/9.594 | 1.861 |
| 29 | 481.35443/9.106 | 1.860 |
| 30 | 144.04556/4.081 | 1.860 |
| 31 | 477.39307/8.445 | 1.856 |
| 32 | 457.36789/10.329 | 1.836 |
| 33 | 545.34723/8.547 | 1.827 |
| 34 | 313.21722/9.18 | 1.778 |
| 35 | 193.10242/5.53 | 1.758 |
| 36 | 265.08749/5.531 | 1.738 |
| 37 | 358.25916/6.955 | 1.703 |
| 38 | 225.07692/3.507 | 1.694 |
| 39 | 215.16576/5.767 | 1.689 |
| 40 | 413.3053/8.341 | 1.671 |
| 41 | 373.20163/7.639 | 1.664 |
| 42 | 169.15991/5.767 | 1.662 |
| 43 | 309.20755/5.865 | 1.611 |
| 44 | 255.12161/4.905 | 1.598 |
| 45 | 187.13432/4.905 | 1.597 |
| 46 | 141.00238/7.595 | 1.583 |
| 47 | 151.04033/3.654 | 1.557 |
| 48 | 281.2489/9.244 | 1.554 |
| 49 | 665.57214/9.356 | 1.529 |
| 50 | 455.10184/1.109 | 1.529 |

**Table S3.** Top 50 features in positive modes classified according to their VIP scores

| **No.** | **Features (*mz*: [M+H]^+^/RT)** | **VIP scores** |
| --- | --- | --- |
| 1 | 206.11487/4.046 | 2.592 |
| 2 | 485.38245/6.004 | 2.500 |
| 3 | 207.17369/6.005 | 2.489 |
| 4 | 189.16277/6.004 | 2.489 |
| 5 | 179.17937/6.004 | 2.471 |
| 6 | 243.19565/6.005 | 2.451 |
| 7 | 265.17712/6.005 | 2.445 |
| 8 | 507.36346/6.004 | 2.442 |
| 9 | 637.38867/8.733 | 2.420 |
| 10 | 220.13071/4.381 | 2.420 |
| 11 | 266.17242/4.666 | 2.416 |
| 12 | 281.1424/6.007 | 2.365 |
| 13 | 383.26126/6.004 | 2.342 |
| 14 | 409.2659/6.587 | 2.339 |
| 15 | 163.14793/6.153 | 2.329 |
| 16 | 397.33002/6.152 | 2.324 |
| 17 | 129.09044/6.153 | 2.322 |
| 18 | 199.16901/6.152 | 2.322 |
| 19 | 181.15848/6.152 | 2.316 |
| 20 | 302.99905/4.704 | 2.314 |
| 21 | 84.95966/4.707 | 2.311 |
| 22 | 321.00946/4.705 | 2.301 |
| 23 | 419.3125/6.153 | 2.291 |
| 24 | 327.14719/6.002 | 2.282 |
| 25 | 234.10933/5.916 | 2.277 |
| 26 | 459.06839/4.703 | 2.277 |
| 27 | 350.25711/8.671 | 2.271 |
| 28 | 241.02948/4.704 | 2.270 |
| 29 | 416.30338/6.154 | 2.263 |
| 30 | 271.09412/7.856 | 2.259 |
| 31 | 259.03983/4.704 | 2.257 |
| 32 | 741.51031/9.825 | 2.256 |
| 33 | 219.04752/4.704 | 2.254 |
| 34 | 193.11955/5.143 | 2.253 |
| 35 | 549.41174/9.386 | 2.251 |
| 36 | 221.1508/6.155 | 2.250 |
| 37 | 189.01544/7.859 | 2.248 |
| 38 | 477.43839/7.73 | 2.248 |
| 39 | 713.47766/9.395 | 2.242 |
| 40 | 248.12498/5.763 | 2.236 |
| 41 | 237.0578/4.702 | 2.223 |
| 42 | 222.14633/7.765 | 2.206 |
| 43 | 353.17145/7.857 | 2.197 |
| 44 | 463.33813/8.554 | 2.185 |
| 45 | 681.50262/7.858 | 2.174 |
| 46 | 214.11987/4.95 | 2.163 |
| 47 | 198.10974/3.55 | 2.162 |
| 48 | 153.12688/5.143 | 2.161 |
| 49 | 171.13736/5.143 | 2.158 |
| 50 | 363.24948/5.143 | 2.157 |

**Table S4.** Main MS/MS fragment ions for compounds annotated at Level 2, compared with reference databases

| **Compound** | **Formula** | **MS** | **MS/MS** | **Database / Reference Spectrum** |
| --- | --- | --- | --- | --- |
| 12-hydroxydodecanoic | C_12_H_24_O_3_ | 216.17303 | 169.1596  213.1494  197.1543 | MassBanl of North America  Spectrum MoNA034669  (last accessed 22/11/2024) |
| δ-dodecalactone | C_12_H_22_O_2_ | 198.16224 | 136.1481  97.1010  121.1010 | MassBanl of North America  Spectrum FIO00053  (last accessed 22/11/2024) |
| 2,4-dodecadienal | C_12_H_20_O | 180.1513 | 81.0701  79.0544  93.0698 | Foodb  *Predicted Spectrum FDB019215  (last accessed 22/11/2024) |

* Note: “Predicted spectrum” refers to in-silico spectra provided in the FooDB entry
